# Dental Infirmary Patients—the Use and Abuse of Dental Charity

**Published:** 1891-09

**Authors:** Richard Grady

**Affiliations:** Baltimore, Md.


### DENTAL INFIRMARY PATIENTS—THE USE AND ABUSE OF DENTAL CHARITY.[1]

BY RICHARD GRADY, M.D., D.D.S., BALTIMORE, MD.

My object in this paper, Mr. President and ladies and gentlemen, is to call attention to the abuse of dental charity, to illustrate its magnitude, and to protest (as every honest and independent man who wishes well to the profession and to its respectability should) against the looseness with which dental colleges dispense their so-called charity,—to "condemn the fault and not the actor of it;" for silence would give an indirect sanction to practices which are lowering the profession in public estimation. So, judging of the proprieties and obligations of my position, I place the statements—dental infirmary patients: the use and abuse of dental charity—together, and propose to consider them in connection, because the evidence in regard to them constantly mingles.

You gentlemen, familiar as you are with recent medical literature, if not appalled at the rapid growth of the dispensary evil, fully realize that it is an evil, that it is taking the means of livelihood from many deserving men, that it is growing, and that it must be combated to be kept within proper bounds.

The sorrows of our cousins across the ocean have been told you in the medical press. "The dispensary abuse in other lands," "the startling excess of medical charity in Edinburgh," "the abnormal growth of the out-patient department at Cambridge," "the great and urgent question of hospital reform" in Birmingham, where the out-patients of hospitals "have advanced in twenty years, 1867 as

---

[1] Read before the Section of Oral and Dental Surgery of the American Medical Association, held in Washington, D. C., May, 1891.

compared with 1887, from 67,000 to 166,000," etc., have been commented upon. "Almost one-half of the people of Edinburgh, Scotland, receive their medical advice gratis. (The population is little over 236,000, and 103,095 dependent on charity for medical advice!) Nor is this all. The number of persons who in any year actually avail themselves of such aid must of necessity be smaller than the total number who would do so were they forced by sickness or accident; and it looks as if more than half the population were in the habit of expecting medical treatment gratis."

In the city of New York wealthy hospital magnates are teaching the people the lessons that they have already learned in Edinburgh. In New York the amount of free work done in hospitals has increased ninety per cent. in the last decade. In time the hospitals may become as much an enemy to the general practitioner as the dispensary is. A Western medical editor, who last summer visited some of the hospitals and clinics of the great metropolis, is quoted in the discussion of the question, "Should a poor man study medicine?" as saying, "What was most striking, outrageous as it is, was the great number of well- and fashionably-dressed, evidently well-to-do people, both male and female, who applied for free treatment. At the ———, on Tuesday, August 12, there were five hundred and sixty-eight patients, and, judging from their appearance, we venture to say that three-fourths at least of them were abundantly able to pay for private medical advice. The profession in New York would be justified," he says, "in arising in emphatic and effectual protest against such wholesale diversion of their revenues."

Why do I recall these things? Not simply to remind you that this co-operation of the medical journals with the medical profession is an excellent example to the dental journals and the dental profession. As far back as March, 1889, I wrote to the *Dental Cosmos* to ascertain if anything on "dental charity" had ever been published. The reply of the editor was, "I do not recall that the subject has been discussed in any dental journal, and I feel quite confident that it has not been in the *Dental Cosmos.* The subject is a delicate one because it concerns a natural but sometimes unprofessional rivalry between dental schools in the same city."

Concerted opposition to dental infirmary abuse has been organized in Baltimore, and the Maryland Dental Protective Association, composed of a majority of the practising dentists in that city, not including those pecuniarily connected with dental schools, came into existence February, 1890; the object of the organization being to regulate the administration of dental charity so that the greatest good may come of it. The members, believing that the impulse must come from the dentists rather than the colleges, are doing what they can to obtain an abatement of the evil which is making such inroads into their practice. Nothing so far has resulted from the movement except the adoption of a resolution,—

"That each of the dental schools be requested to place in the infirmaries (near the entrance) record-books in which shall be printed in bold type on each page, 'For the poor only,' and in which shall be entered the name and address of all applicants for dental services. After such registration each applicant shall receive a card also headed 'For the poor only,' if he or she admits inability to pay the usual office fees; all others shall be rigidly excluded from infirmary privileges. This card shall be retained by the patient and presented at each visit, and until the completion of all necessary services, when the card (endorsed by the operating student with his statement) shall be returned to the demonstrator and placed on file. The record-books and the cards shall be open for public inspection at all reasonable times."

The president of the Association has also visited the mayor of the city to request that no appropriations be made the dental schools for 1891 unless in the contract there be included a provision that (1) all applicants for dental services shall be registered, and (2) that the record-books be open for inspection. Appropriations of four hundred and five hundred dollars had been made in 1888 and 1889 to one of the dental schools "for supplying medicines to the indigent sick of the city of Baltimore."

In what follows I wish it to be understood that I have no reference to any special college. My remarks are addressed to the system generally. Many of the facts are calculated to arrest the attention and engage the study of the best men in the profession.

Believing that every reform was once a private opinion, and, acting upon the advice of Emerson that "that statement only is fit to be made public which you have come at in attempting to satisfy your own curiosity," I, judging it expedient to gather light from quarters deemed trustworthy, wrote to deans of dental schools to determine whether dental college infirmaries truly deserve the designation of charities. The general result of the inquiry confirmed the impression that there is an enormous abuse of so-called dental charity by the almost reckless admission of all comers, and that the practice of some colleges is large and pecuniarily profitable.

As no man's personal rights are in the issue, I give you some of the information elicited as to infirmary charges and infirmary practice:

1. "Our infirmary privileges are extended only to those unable to pay the usual office fees (all others are rigidly excluded); gold fillings, seventy-five cents to two dollars and fifty cents; amalgam and other plastics, twenty-five cents to one dollar; vulcanite (full upper or lower), five dollars and fifty cents; gold or continuous gum, twenty-five dollars; no record of 'charity patients' to whom relief is extended without fee other than extractions and pathological cases."

2. "The charge made is intended to cover actual expense of materials used and whatever may be used in a charitable way; there is usually a surplus of receipts over expenditures; minimum charge for filling with gold, fifty cents; for dentures, six dollars; these amounts vary, of course, if a large quantity of material is used or in the case of dentures being made on metal bases."

3. "The dental college charges simply for material used in plugs and dentures, and tries to avoid doing for people who are able to pay regular dentists."

4. "We average about eight thousand patients of all kinds yearly; charge for gold fillings and other work about what material costs, estimating rather over than under, as we can't afford to lose by our good works."

5. "The fund we collect from our clinics amounts to about five thousand dollars per year of ten months, while we are in opertion."

6. "Charges are slightly more than cost of material; we aim to have the infirmary self-supporting."

7. "We try to have the charges for material used cover the waste and as much of the running expenses of the infirmary as practicable."

8. "Gold for about one thousand patients, for which a profit is charged; make no distinction in patients."

9. "No regular tariff for services; endeavor to discourage well-to-do by making charges higher than in office."

10. "Charge for gold filling about one-half what dentists charge."

11. "Our purpose is to make infirmary self-sustaining, etc."

The true work—the only legitimate work—of dental colleges should be to educate students for professional life, to perfect the professional education of the dentist. But this preliminary training should not be used, as it sometimes is, to lower the dignity or emoluments of private practice. For pecuniary interest, some Faculties attract as many students as possible (over three thousand one hundred were in attendance at the various dental colleges of the country for the session 1890–91), whom they send forth in the spirit of rivalry into the ranks of the profession without a thought that the means for subsistence for these men is diminished by dental infirmaries taking patients which these graduates should have, that having raised up these men to professional life they have the further duty not to pull them down, and that they are warring upon the livelihood of their professional brethren. Is it any wonder, then, that men often lose heart after struggling for a time against so powerful and so unscrupulous a rival as every dental college infirmary is, whose anxiety for abundance of clinical material is so pressing that there is no discrimination, or hardly any? and that men of talent and respectable professional ability are forced to adopt rates that will in a measure compete with clinical fees in order to secure at least a moderate share of practice, or be driven out of the profession to find support in some other occupation? Have members of the dental profession no feelings which influential dental colleges are bound to respect?

More than fifty years ago, as you know, the *first* dental college was founded. It was the purpose to give the institution a high character at the start. After it had become necessary to establish an operating infirmary, "for the benefit of the students," this announcement was published:

"There exists abundant opportunity for practical instruction in the dental infirmary attached to the institution. The appreciation and consequent demand of such operations as are here performed are steadily increasing among the indigent of our city. It may be necessary to add that these operations are not performed by the professors in the presence of the class, but by the students themselves under the immediate eye and direction of their instructors. In fine, the object of the institution is to perfect the professional education of the dentist, which may or may not have been begun under some competent private instructor, and which, it is recommended, should be pursued during the summer vacation in some dental office."

Has the stamp of character the infirmary then received endured?

The origin of the dental infirmary has been told you. The evolution of the abuse can readily be traced.

1. "Operations among the indigent." "All expenses borne by college."

2. "Students have the privilege of operating upon their own private patients."

3. The natural development has been that services have been rendered infirmary patients who could pay a proper fee; that "during the sessions the operative and mechanical departments are now so well patronized that every student can obtain as much practice as is possible for him to attend to;" that "the clinical material is abundant, there being an excess of patients;" that "the practice for dental students has increased to such an extent that all the students during the past session have had an abundance of practical work in both operative and prosthetic dentistry,—the record-books showing to the credit of many of them hundreds of gold fillings inserted for infirmary patients, besides other operations. This means for practical instruction has already assumed such large proportions that the supply has been beyond the needs of the large classes in attendance during the past sessions."

That the competition of some dental college infirmaries affects to an appreciable degree dentists injuriously will be obvious to every reflecting person at the bare mention that "about twenty-five thousand patients are attended to yearly" by one dental college, and "on an average for nine months in the year relief is extended to about one hundred patients a day" by another; that one dental college which was "extending relief to more than two thousand charity patients" in 1889 reported next year (1890), "more than ten thousand of such cases," and another which reported in 1889 "the charity patients number four thousand three hundred and twelve," in 1891 (two years later) gave the number of cases "twelve or fifteen thousand."

This competition is most onerous to the dentists of least income, and to the practitioners who are struggling against the odds of cheap dentistry in an effort to maintain an honorable position and eke out of their labors sufficient income for a respectable living. The dental infirmaries being open to all classes and conditions of men and women who desire low-priced services, few, if any, questions are asked so long as the charges are paid, no matter how elegant and costly the habit nor how many jewels displayed. This pernicious example of cheap dentistry is an injustice to those dentists who resist mercenary competition and endeavor to render their bills in conformity with standard fees.

Are there not poor enough to supply "clinical patients" without encouraging the well-to-do to degrade the profession by responding to the enticing and seductive advertisement, "Many of the operations are performed free of charge, and all others at the cost of materials only," or the knowledge that "during the first two months of the session we furnish all materials to patients free of cost," although it may be said in excuse of the work done for them, "the operations are faulty and but the handiwork of students?" Is it not true benevolence and sound policy to remove, if possible, competition from those least able to bear it? If fair-minded men think so, if the Faculties of dental schools think so, then let the welfare of the whole profession supersede and extinguish the selfishness of the few.

A dentist who would endeavor to obtain practice after the model of the pretentious dental infirmary announcement—"free to all—all operations known to modern dentistry performed in most workmanlike manner; gold-foil and artificial teeth at actual cost; amalgam or silver, white, tin, and gutta-percha fillings free; this is the first time such an opportunity has ever been presented to every one to save those important organs (the teeth) free of cost; old and experienced demonstrators in charge; the same decorum will be observed as in private office, etc., etc."—would be accused of unprofessional conduct, of acts unworthy the lowest grades of the profession, and set down among his fellows as unprincipled, a professional outlaw fit to "send to Coventry."

However painful it may be to admit, it cannot be denied that methods which are legitimate for a dental college are not improper for the individual dentist, although claimants for public patronage may not see the question in this equivocal position after they have worked themselves into dental college Faculties.

But the effects of the practice are not worse than the statements in the advertisements. One of the most pregnant counts in the indictment against the dental college infirmaries is that they advertise "gold-foil and artificial teeth at actual cost." Dental colleges should give an equivalent for the fees they demand: to do *more* is charity, to do *less* is fraud.

Lest it should be thought that I am making statements to suit my argument, let me quote what those think who ought to know best, from the opportunities of observation and experience. The views expressed by the gentlemen are creditable to their sense of justice, and will have such weight with the membership of this association and with others as they are entitled to.

Dr. Shepard, president of the Massachusetts Board of Registration, whose caustic criticisms are fresh in the minds of readers of dental literature, says, "Suppose one college furnishes only No. 3 gold in the infirmary, and sells each sheet for seventy cents, making a profit of sixty dollars per ounce, and another college furnishes No. 4 gold, and sells it at forty cents a sheet, making a profit of fourteen dollars and forty cents per ounce, would it not be reasonable to infer that the former was run more for profit to the college and the latter more for the advantage of the student?"

The testimony of Dr. Genese, who holds official position in two dental schools, is: "It is well known that persons amply able to pay (occasionally persons of known wealth) claim the attention of the students, and needed charity kept so long waiting that they depart or do not get the work done, as the charges are sometimes as much as a young practitioner will make. Where is the charity? and how does the dental infirmary compare this with only 'cost of material'?

"No student should be allowed to appoint time for people to visit him at the institutions. Every patient should be first seen by the demonstrator and the work given in rotation to students. The name and address and occupation of the applicant should be obtained, and if found able to pay should be referred to a dentist, and not, as at present, charged full fees to go into the pockets of the professors and demonstrators."

Dr. Ferdinand Groshans, a demonstrator is of this belief: "From personal experience at the dental infirmary as student and assistant I could cite cases of patients who were able to pay and had their teeth filled and plates inserted to their own disadvantage as well as to the injury of the science of dentistry, both of which are due in my opinion to the want of a larger number of instructors as well as to the inexperience of the new beginner. I think a less number of patients would be sufficient and of more value to the student if the instruction would be more of a personal supervision and more constant by the demonstrators in charge. I do not think a student should be left so much to himself as is the practice at present. The infirmary practice as at present conducted is detrimental to the patient, student, college, and the profession in particular, to say the least, and not considering the practitioner who must live by the legitimate practice of his profession." These views a vice-president of the National Association of Charities approved, and added, "We want fewer unworthy applicants and a better service in the dispensaries."

Dr. J. L. Asay, of California, writing on "the pernicious effect of dental college clinics," says, "That such a condition exists was to me made apparent during my visit to the larger cities of the Atlantic coast last year, but was not confined to any particular dental school. I found it, however, to predominate in Philadelphia, where there are no less than three dental colleges. It may possibly be that I observed this state of affairs more in that city than in other places from the fact of my larger acquaintance there than elsewhere, it being my native city and former residence for twenty-seven years; hence other localities may furnish equal grounds for comment.

"During the visit to which I have alluded, I frequently heard old practitioners complain of college operations and their fees. I heard men and women of good circumstances and comfortable incomes congratulate themselves and each other upon how cheap they had got their teeth filled or plates made at the dental colleges. We may rail against gas-offices and bucket-shops, five-dollar sets of teeth, and 'teeth plugged to last for life for twenty-five cents' without avail, so long as we countenance the pernicious example of cheap dentistry and low fees by our institutions of dental learning to those who should be compelled to seek the services of the regular practitioner, and pay a price equivalent to the time occupied and the skill employed."

This expression is confirmed by an official in Philadelphia under date of April 30, 1891, who writes: "In this city, with its three large dental schools (having in attendance over seven hundred students during the last session) actively competing for patronage, the evils of indiscriminate charity are seriously felt by a large number of worthy practitioners. Such charity certainly tends to cheapen the services of the dentist and in many cases causes young graduates to resort to questionable methods of obtaining a remunerative practice. The question is of vital and timely importance."

In conclusion, what is the duty of the hour? What, it may be asked, do members of the dental profession propose for the evil which they allege to exist so much to their prejudice and detriment? Is it to close the dental infirmaries? Not at all, xcept, possibly, during the vacation when no lectures are delivered; because in our present social condition the dental infirmary may be necessary (I will not say necessary evil). They suggest a cure more humane, more salutary, more manly, more worthy of them. It is that dental infirmary privileges be extended only to those unable to pay the usual fees of dentists, because it is believed that the practice of dental schools in throwing open their doors to whoever may apply injuriously affects both the public and the dental profession. (The testimony upon this point is so honorable to the dentists of Baltimore that special attention is invited to it, as it has been affirmed and re-affirmed by members of the Maryland Dental Association, and is in fact the sentiment that led to the organization.)

I suggest the following for consideration as possible remedies; other thinkers may be stimulated to supplement what has been omitted. Oneness of aim does not imply identity of means.

1. Go back to the practice of the time-honored pioneer: an operating infirmary "for the benefit of the students," operations "among the indigent" only "by the students under the immediate *eye* and direction of their instructors," "all expenses borne by college."

2. Let no "students have the privilege of operating upon their own patients (private) during the sessions," either at the college or at their homes.

3. Provide for dental infirmary privileges to persons recommended by dentists or by some inquiry agency familiar with the circumstances of applicants, similar to the Charity Organization Society of Baltimore.

These are simple suggestions, and it only remains for the dental colleges, more especially rival institutions in the same city, or those which "aim to have the infirmary self-supporting," or have a "surplus of receipts over expenditures," to put them in practice. Even if it had been found difficult to devise remedies, the calling of the attention of the public to the evil would have been useful.

The interests of the dental profession and of the dental colleges are really identical, and they should be made practically so. At present there is a combat between those with whom unanimity of purpose and honesty of intention should prevail. Each sets the other at defiance, and as a rule there is little kindly feeling and few friendly acts on either side. It would be otherwise if the graduates of dental schools, instead of having to suffer from acts prejudicial, or sometimes injurious, to their interests were made the objects of a generous parental care. Such consideration would dispel antagonism, and the charity of the public could be confidently appealed to with the support of the profession, notwithstanding there would be (apparently) a falling off in the number of patients at the dental infirmaries.

This is an age of associations, hence it is desirable that State societies be formed which shall co-operate in protesting emphatically against the abuse of dental charity and in taking effectual measures for its correction, so that in case of application for State or municipal (financial) aid on the plea that "the Faculty are receiving now scarcely any remuneration, they ask that they be relieved from the cost of the very charitable institution which they have necessarily connected with the college;" or "the Faculty find themselves compelled to ask aid from the State; they are not willing that the institution shall perish without giving the State an opportunity to preserve it," it may be made known that no such assistance is given in the cities of Boston, New York, Philadelphia, Washington, Chicago, Indianapolis, Cincinnati, Louisville, Minneapolis, or San Francisco by the tax-payers to dental schools.

